# JAK2V617F and p53 mutations coexist in erythroleukemia and megakaryoblastic leukemic cell lines

**DOI:** 10.1186/2162-3619-1-15

**Published:** 2012-06-21

**Authors:** Wanke Zhao, Yanhong Du, Wanting Tina Ho, Xueqi Fu, Zhizhuang Joe Zhao

**Affiliations:** 1 Department of Pathology, University of Oklahoma Health Sciences Center, Oklahoma City, OK, 73104, USA; 2 Edmond H. Fischer Signal Transduction Laboratory, College of Life Sciences, Jilin University, Changchun, China

**Keywords:** JAK2, TP53, Leukemia, Mutations, Transformation

## Abstract

**Background:**

JAK2V617F, a gain-of-function mutant form of tyrosine kinase JAK2, is found in the majority of patients with Ph- myeloproliferative neoplasms (MPNs), a group of chronic hematological diseases that often lead to acute leukemia. The current study is intended to find other gene mutations that collaborate with JAK2V617F to cause leukemic transformation.

**Methods:**

Total RNA and genomic DNA were isolated from two JAK2V617F-positive cell lines, namely, erythroleukemic HEL and megakaryoblastic leukemic SET-2 cells. Candidate genes were amplified by PCR and further sequenced.

**Results:**

Homozygous mutations of the *TP53* gene which encodes tumor suppressor p53 were found in HEL and SET-2 cells. While HEL cells, which have homozygous JAK2V617F, contain a rare M133K p53 mutation, SET-2 cells, which have a heterozygous JAK2V617F mutation, contain a common R248W p53 alteration. Western blot analyses revealed high levels of p53 expression in both cells. M133K and R248W are located in the DNA binding domain of p53. Structural analyses revealed that they potentially disrupt the interaction of p53 with DNA, thereby causing loss of p53 function.

**Conclusions:**

JAK2V617F and p53 mutations coexist in leukemia cells. We believe that JAK2V617F is able to drive leukemic transformation when the function of tumor suppressor p53 is lost. The interplay of JAK2V617F with p53 may affect the progression of MPNs.

## Background

Ph- myeloproliferative neoplasms (MPNs) are clonal hematopoietic disorders in which one or more myeloid lineages are abnormally amplified. These diseases represent a group of chronic conditions including polycythemia vera (PV), essential thrombocythemia (ET), and primary myelofibrosis (PMF) [1]. MPNs mainly affect older people with an average age of onset of 55 years. Complications associated with MPNs include development of acute leukemia as well as thrombosis, hemorrhage, and myeloid metaplasia. The major molecular lesion in these diseases is JAK2V617F, which occurs in over 90% of PV and over 50% of ET and PMF [1,2]. JAK2V617F has enhanced tyrosine kinase activity, and it causes constitutive activation of down-stream signal transducers when expressed in cells [3]. Studies have demonstrated that transgenic expression or knock-in of JAK2V617F in mice causes MPN-like phenotypes [4,5]. However, whether or not JAK2V617F is able to drive leukemic transformation is not known.

Malignant transformation usually involves a gain-of-function mutation of oncogenes and a loss-of-function mutation of tumor suppressor genes. Among various tumor suppressors, p53, which is encoded by the *TP53* gene, has been extensively studied [6-8]. *TP53* is mutated or inactivated in over 60% of cancers. Normal p53 suppresses malignant transformation by controlling cell cycle progression, ensuring the fidelity of DNA replication and chromosomal segregation, and inducing apoptosis in response to potentially deleterious events. Interestingly, mutations of p53 are the most common in solid tumors but relatively rare in blood cell malignancies such as leukemia [7,9]. In this study, we investigated the mutation status of p53 in two JAK2V617F-positive leukemic cell lines. We found mutations of p53 in both cells. One cell line bears a rare M133K mutation, while the other contains a R248W alteration frequently found in other tumors.

## Results and discussion

### Identification of p53 mutations in HEL and SET-2 cells

**Figure 1:**
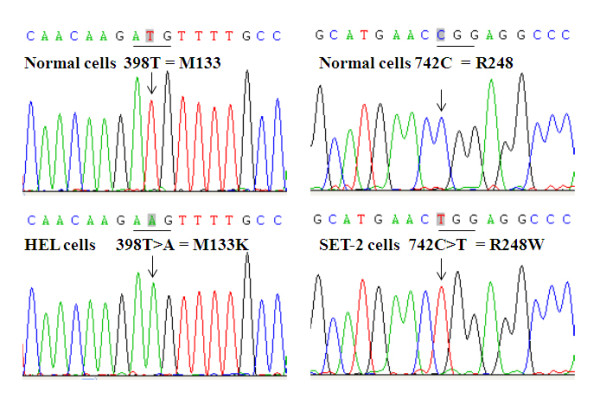
**Identification of p53 mutation in HEL and SET-2 cells.** The entire coding sequence of p53 was amplified by reverse transcription PCR from total RNAs isolated from HEL and SET2 cells together with a normal white blood cell sample for comparison. The positions of the mutation sites are indicated by arrows, and the associated changes in codons and amino acids are shown. Note that the p53 mutations found in HEL and SET2 cells are homozygous. DNA sequencing was also performed from the reverse direction with consistent results (not shown).

The constitutive activation nature of JAK2V617F makes it a potential oncoprotein. To identify other gene mutations that collaborate with JAK2V617F to drive leukemia cell transformation, we employed two well-studied leukemia cell lines, HEL and SET2, which are known to contain JAK2V617F [10]. HEL (ATCC no. TIB-180) was derived from an erythroleukemia patient [11], and SET-2 (DSMZ no. ACC 608) cells were established from an ET patient at megakaryoblastic leukemic transformation [12]. To investigate the mutation status of p53, we isolated total RNA from the cells. Following reverse transcription, the entire coding sequence of p53 was amplified by PCR. Complete DNA sequencing analyses revealed two distinct gene mutations in HEL and SET-2 cells (Figure 1). In HEL cells, the mutation is a T-to-A transversion at position 398 of p53 cDNA, resulting in an M-to-K substitution at amino acid residue 133 of the p53 protein. In SET-2 cells, the mutation is a 742C-to-T transition at the DNA level resulting in a R248W replacement at the protein level. Note that both mutations are homozygous. We also verified the mutations by amplifying genomic DNAs. The p53 mutations are found in exon 5 and 7 of the *TP53* genes in HEL and SET-2 cells, respectively. DNA sequencing analyses of PCR products containing the correspondent exons revealed homozygous mutations of p53 at the gDNA level (data not shown). This indicates the homozygosity is likely caused by mitotic homologous recombination rather than gene silencing at the epigenetic level.

**Figure 2:**
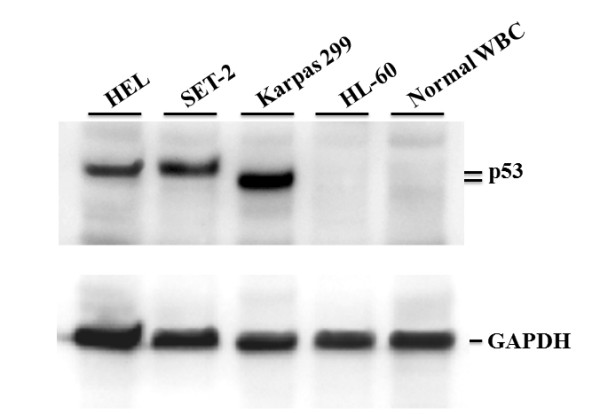
**Expression levels of p53 in HEL and SET-2 cells.** Whole cell extracts of HEL, SET-2, Karpas 299, HL-60, and total normal white blood cells were subjected to Western blot analyses with a pantropic anti-p53 (Ab-2) antibody. Protein loadings were revealed by an antibody against glyceraldehyde 3-phosphate dehydrogenase (GAPDH). Note that p53 in Karpas 299 cells runs slightly faster on the gel.

Protein expression levels of p53 in HEL and SET-2 cells were revealed by Western blot analyses (Figure 2). Robust expressions of p53 were found in both cells as in T cell lymphoma Karpas 299 cells which contain an R273C mutation [7]. In contrast, p53 is absent in promyelocytic leukaemia HL-60 cells which are known to contain a *TP53* gene deletion [7]. Likewise, expression of p53 was hardly detectable in white blood cells from a normal blood sample. These results support the notion that M133K and R248W are loss-of-function mutants. The expression level of normal p53 is kept low through a continuous degradation mediated by MDM2, which is itself a product of p53-activated gene expression, while mutant p53 proteins which lose transcription activity do not induce MDM2 and are thus able to accumulate at very high concentrations [6-9].

**Figure 3:**
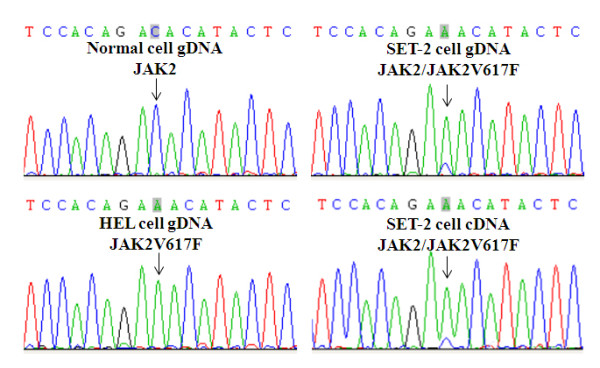
**Verification of the presence of JAK2V617F in HEL and SET-2 cells.** DNA fragments containing JAK2 exon 14 and full-length JAK2 cDNA were amplified by PCR from genomic DNAs and reverse-transcribed single-strand cDNAs, respectively. DNA sequencing was performed from the reverse direction. Positions of the V617F mutation sites are indicated by arrows. Note that HEL cells carry a homozygous mutation while SET2 cells contain a heterozygous mutation at both genomic DNA and cDNA levels. DNA sequencing was also performed from the forward direction with consistent results (not shown).

We then verified the mutation status of JAK2 in HEL and SET-2 cells used in our study. The presence of JAK2V617F was readily detected by using a simple allele-specific PCR technique [13] (data not shown). We further amplified a DNA fragment covering the exon 14 of JAK2 by PCR with genomic DNA as templates. DNA sequencing analyses revealed a homozygous mutation of JAK2 in HEL cells and a heterozygous JAK2 mutation in SET-2 cells (Figure 3). To see if both mutant and wild type alleles are expressed in SET-2 cells, the full-length coding sequence of JAK2 was amplified from total RNA by reverse transcription PCR. Sequencing of the RT*-*PCR products demonstrated heterozygous JAK2 mutation, indicating expression of both wild and mutant alleles in SET-2 cells (Figure 3). DNA sequencing data also revealed the predominant presence of JAK2V617F in SET-2 cells. By using a real time PCR method previously described [14], we determined that the ratio of JAK2V617F to JAK2 is approximately 6:1 in SET 2 cells at both the genomic and cDNA levels. Apparently, mutation of JAK2 in SET-2 is also accompanied by gene amplification of the mutant allele. It should be noted that SET-2 cells were derived from an ET patient. It is generally believed that ET patients usually bear a heterozygous JAK2 mutation while PV patients mostly contain homozygous JAK2V617F. Our data suggest that in the heterozygous cases, the ratio of JAK2 and JAK2V617F may not be necessarily equal.

### Structural analysis of p53 mutants found in HEL and SET-2 cells

**Figure 4:**
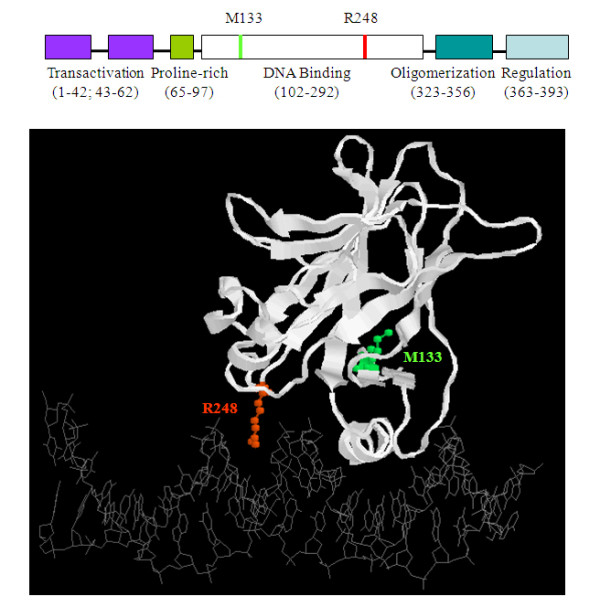
**Potential deleterious effects of the M133K and R248W mutations on structure and function of p53.** The upper panel shows a schematic diagram of p53 structure with the relative positions of the M133K and R248W mutation sites indicated. The lower panel demonstrates the crystal structure of the DNA binding domain of p53 in complex with DNA. Side chains for M133 (green) and R248 (red) are shown as balls and sticks, while the rest of the amino acid residues are represented in the backbone by ribbons.

We thus identified two distinct p53 mutations in JAK2V617F-positive HEL and SET-2 cells. While the M133K mutation identified in HEL cells is rare, R248W found in SET-2 cells is one of the most frequently mutated residues of p53 in human cancers [7]. According to the IARC TP53 database (Version R15, November 2010, see ref. 7), M133K has been found as a somatic mutation in 20 tumors, whereas R248W has been reported as a somatic mutation in 733 tumors and as a germline mutation in 18 families with Li-Fraumeni syndromes. The functional consequence of R248W alteration has been extensively studied, but there is essentially no reported study on the M133K mutant [7]. Both M133K and R248W are located in the DNA binding domain of the p53 molecules. Based on the crystal structure of the DNA binding domain of p53 in complex with DNA [15], structure analyses predict that both mutations have deleterious effects on p53 function (Figure 4). The R248 residue forms loop 3 of the p53 core structure and reaches into the minor groove of DNA. It presumably plays a critical role in DNA binding, and mutation of this basic residue to a bulky aromatic tryptophan residue should have detrimental effects on binding of p53 with DNA. The M133 residue, on the other hand, is located in the S2' strand and is buried in the loop-sheet-helix motif. This motif makes up the major DNA binding surface of the p53 molecule. Although M133 does not directly interact with DNA, its mutation to a basic arginine residue may significantly alter the conformation of the entire loop-sheet-helix motif, thereby disrupting DNA binding.

The mutation rate of p53 in leukemia overall is very low, but it is much higher in acute myeloid leukemia with a complex aberrant karyotype [7,9,16]. Interestingly, both HEL and SET-2 cells display an abnormal karyotype with selective JAK2 chromosomal amplification and concomitant deletion of the residual homolog [10]. Indeed, the 6:1 JAK2V617F/JAK2 ratio found in our current study suggests amplification of the mutant JAK2 allele. In addition, earlier studies identified p53 mutations in about half of MPN patients in blast crisis [17,18], suggesting inactivation of p53 may be relatively frequent in blastic transformation of MPNs and is required for the process. These studies were conducted before the discovery of JAK2V617F but included analysis of NRAS and KRAS which were found intact. Our current study demonstrates coexistence of JAK2V617F and mutations of p53 in leukemia cells. This provides evidence that JAK2V617F likely drives leukemic transformation when p53 function is lost. We also believe that the dependence of MPN phenotypes on aging may be related to decreasing activity of p53. Among various factors affecting aging, p53 is most extensively studied [19-21]. In fact, loss of p53 activity is considered an aging process. Besides p53 mutations, p53 function can also be inactivated by other mechanisms. For example, a recent study demonstrated that JAK2V617F negatively regulates p53 stabilization by enhancing MDM2 via La expression in MPNs [22]. Our data further support the notion that JAK2V617F interacts with p53 to promote progression of MPNs. Therefore, inhibiting JAK2V617F and maintaining p53 function are of utmost therapeutic importance.

## Conclusions

We identified the coexistence of JAK2V617F and p53 mutations in leukemia cells. This suggests that JAK2V617F is able to drive leukemic transformation when the tumor suppressor function of p53 is lost. Decreased p53 activity as a consequence of aging may also affect the progression of MPNs. Understanding the interplay of JAK2V617F and p53 should have major implications for prevention and treatment of the diseases.

## Methods

### Cells and DNAs

HEL (ATCC no. TIB-180) and SET-2 (DSMZ no. ACC 608) cell lines were purchased from American Type Culture Collection **(**ATCC**)** and German Collection of Microorganisms and Cell Cultures (DSMZ), respectively. The cells were maintained in RPMI 1640 medium supplemented with 20% heat-inactivated fetal bovine serum. White blood cells were isolated from de-identified normal blood samples upon lysis of red blood cells. Total RNAs were isolated from cells by using the Trizol reagent (Invitrogen), and single strand cDNAs were synthesized with random primers by using a reverse transcription kit from Promega. Genomic DNAs were purified by using the phenol/chloroform method after proteinase K digestion of whole cell lysates.

### PCR and sequencing analysis

PCR was run with high fidelity Phusion DNA polymerase. The entire coding regions of p53 and JAK2 were amplified from single strand cDNAs. The PCR primers used were GCCAGACTGCCTTCCGGGTCACT (forward) and AGAGATGGGGGTGGGAGGCTGTC (reverse) for p53, and TGCATGGGAATGGCCTGCCTTAC (forward) and CTTTCATCCAGCCATGTTATCCCTTA (reverse) for JAK2. Genomic DNA surrounding the entire exon 14 of the *JAK2* gene was amplified by PCR from genomic DNAs with primers GATCTCCATATTCCAGGCTTACACA (forward) and TATTGTTTGGGCATTGTAACCTTCT (reverse). The PCR products were gel-purified and subjected to DNA sequencing analyses by using an ABI 3730 capillary sequencer at the core facility of University of Oklahoma Health Sciences Center. DNA sequencings were performed from both forward and reserve directions. To determine the ratio of JAK2 and JAK2V617F at the genomic or cDNA level, real time PCR analyses were performed as previously described [14], except that purified plasmid DNAs were used as standards [13].

### Western blot analyses

Cells were extracted in SDS gel sample buffer. Upon separation on 10% SDS gels and transferring to PVDF membranes, proteins were probed with antibodies against p53 and glyceraldehyde 3-phosphate dehydrogenase followed by a horseradish peroxidase-conjugated secondary antibody. Detection by the electrochemiluminescence method and capture of immunoblot images were carried out by using FluorChem SP imaging system from Alpha Innotech as previously described [23].

## Abbreviations

MPN: Myeloproliferative neoplasms; PV: Polycythemia vera; ET: Essential thrombocythemia; PMF: Primary myelofibrosis; PCR: Polymerase chain reaction.

## Competing interests

The authors declare no conflict of interests.

## Authors’ contributions

WZ, YD, and WTH performed the research experiments. XF designed the research and analyzed the research data. ZJZ designed the research and wrote the manuscript. All authors read and approved the manuscript.
